# Long-Term Exposure to Nanosized TiO_2_ Triggers Stress Responses and Cell Death Pathways in Pulmonary Epithelial Cells

**DOI:** 10.3390/ijms22105349

**Published:** 2021-05-19

**Authors:** Mayes Alswady-Hoff, Johanna Samulin Erdem, Santosh Phuyal, Oskar Knittelfelder, Animesh Sharma, Davi de Miranda Fonseca, Øivind Skare, Geir Slupphaug, Shanbeh Zienolddiny

**Affiliations:** 1 National Institute of Occupational Health, NO-0033 Oslo, Norway; mayes.alswady-hoff@stami.no (M.A.-H.); Johanna.Samulin-Erdem@stami.no (J.S.E.); santosh.phuyal@medisin.uio.no (S.P.); Oivind.Skare@stami.no (Ø.S.); 2 Department of Molecular Medicine, Institute of Basic Medical Sciences, University of Oslo, NO-0316 Oslo, Norway; 3 Max Planck Institute for Cell Biology and Genetics, 01307 Dresden, Germany; knittelf@mpi-cbg.de; 4 Department of Cancer Research and Molecular Medicine, Norwegian University of Science and Technology, NO-7491 Trondheim, Norway; animesh.sharma@ntnu.no (A.S.); davi.fonseca@ntnu.no (D.d.M.F.); geir.slupphaug@ntnu.no (G.S.); 5 Proteomics and Metabolomics Core Facility (PROMEC), Norwegian University of Science and Technology and the Central Norway Regional Health Authority, NO-7491 Trondheim, Norway

**Keywords:** titanium dioxide, fibrosis, carcinogenesis, proteomics, lipidomics

## Abstract

There is little in vitro data available on long-term effects of TiO_2_ exposure. Such data are important for improving the understanding of underlying mechanisms of adverse health effects of TiO_2_. Here, we exposed pulmonary epithelial cells to two doses (0.96 and 1.92 µg/cm^2^) of TiO_2_ for 13 weeks and effects on cell cycle and cell death mechanisms, i.e., apoptosis and autophagy were determined after 4, 8 and 13 weeks of exposure. Changes in telomere length, cellular protein levels and lipid classes were also analyzed at 13 weeks of exposure. We observed that the TiO_2_ exposure increased the fraction of cells in G1-phase and reduced the fraction of cells in G2-phase, which was accompanied by an increase in the fraction of late apoptotic/necrotic cells. This corresponded with an induced expression of key apoptotic proteins i.e., BAD and BAX, and an accumulation of several lipid classes involved in cellular stress and apoptosis. These findings were further supported by quantitative proteome profiling data showing an increase in proteins involved in cell stress and genomic maintenance pathways following TiO_2_ exposure. Altogether, we suggest that cell stress response and cell death pathways may be important molecular events in long-term health effects of TiO_2_.

## 1. Introduction

Titanium dioxide (TiO_2_) nanomaterials are utilized in a wide range of products including paints, coatings, plastics, pharmaceuticals, cosmetics, and food [1]. The biodurability and increased use of nanosized TiO_2_ materials in industrial production raise concerns about potential adverse health effects among exposed workers. Exposure to TiO_2_ may occur during manufacturing, use, and waste handling, and lead to accumulation of ultrafine TiO_2_ particles in the lungs, as reviewed by Shi, et al. [2]. Epidemiological data on TiO_2_ exposure by inhalation are limited and clear evidence of adverse health effects are lacking [3]. However, in exposed workers, a reduced lung function [3] and a correlation between levels of exposure and changes in biological responses, e.g., increased levels of oxidative biomarkers, have been observed [4,5,6,7]. Several studies in animals indicate that TiO_2_ exposure causes lung inflammation, fibrosis, and cancer [8,9,10]. TiO_2_ exposure by inhalation or intratracheal instillation may lead to pulmonary inflammation, accompanied by histological changes indicative of lung fibrosis and increased infiltration of inflammatory cells, as well as induced serum levels of proinflammatory cytokines in experimental animals [11,12,13,14]. Furthermore, development of TiO_2_-induced pulmonary inflammation may involve IL1 receptor and IL1-alpha signaling events similar to those observed for asbestos [15]. Interestingly, some studies indicate that the inflammatory response is transient and alleviated after prolonged exposure [13]. However, rats exposed to high doses of TiO_2_ for two years develop lung tumors [16,17], and TiO_2_ have been classified as possibly carcinogenic to humans (Group 2B) by the International Agency for Research on Cancer [18]. Moreover, the European commission (ECHA) classified the TiO_2_ as a category 2 carcinogen [19].

Several in vivo and in vitro studies have addressed the molecular mechanisms underlying the potential genotoxic and carcinogenic effects of TiO_2_ exposure, with varying results [2]. Short-term studies on pulmonary epithelial cells have demonstrated increased DNA damage, oxidative stress, ROS generation, and structural chromosomal aberrations [20,21]. Titanium dioxide exposure affects the expression of proteins involved in a wide range of cellular stress responses including oxidative and DNA replication stress, metabolism, adhesion, cytoskeleton remodeling, cell growth, apoptosis, and cell cycle arrest [22,23,24,25]. The same studies have shown that genomic instability occurs already after 24 h of exposure. Long-term exposure to TiO_2_ induces cell transformation and altered expression of proteins regulating mitochondrial function, cellular trafficking, and proteasome activity [26,27,28]. Prolonged exposure also causes more severe DNA damage than acute exposure [29]. Nevertheless, some studies show no effects of TiO_2_ exposure on cellular toxicity or DNA damage [30,31].

Most in vitro studies addressing possible health hazards of nanomaterials have been based on short-term exposure (24–72 h) with relatively high concentrations (up to 200 µg/mL). Further in-depth studies of cellular responses to long-term and low-dose TiO_2_ exposure are thus warranted. To investigate potential molecular mechanisms underlying adverse health effects of long-term TiO_2_ exposure, this study focuses on assessing effects of TiO_2_ on key cellular pathways such as cell cycle, apoptosis and autophagy, as well as proteome and lipidome profiles of exposed pulmonary epithelial cells.

## 2. Results

### 2.1. Characterization of TiO_2_ Particles

**Figure 1:**
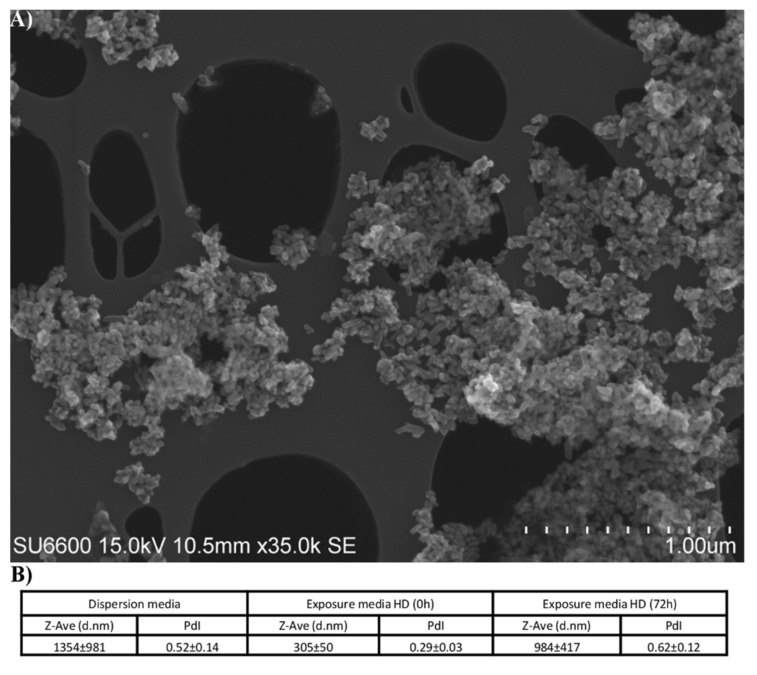
Characterization of TiO_2_ particles. (**A**) Representative SEM image of TiO_2_ particles in dispersion media. (**B**) Hydrodynamic diameter measurements were conducted in dispersion media, and in cell culture media at time of exposure (0 h) and after 72 h. Abbreviations: Z-Ave: Z-average; d.nm: diameter in nm; PdI: polydispersity index.

The TiO_2_ nanomaterials were characterized using SEM, DLS and endotoxin levels were analyzed by LAL assay. SEM micrographs in Figure 1A showed that single TiO_2_ particles were spherical, and that the majority of the particles were found as aggregates or agglomerates of varying sizes. This is in accordance with the size distribution of TiO_2_ particles measured by DLS (Figure 1B) which showed a high degree of instability and batch-to-batch variation with hydrodynamic diameter (Z-Ave) of 1354 ± 981 nm in dispersion media. DLS measurements of TiO_2_ in exposure media showed a hydrodynamic diameter of 305 ± 50 at the start of the exposure (0 h), while after 72 h of exposure the hydrodynamic diameter increased to 984 ± 417 nm, indicating a tendency to aggregation and agglomeration over time. Endotoxin levels of the TiO_2_ nanomaterials were below the detection limit of 0.005 EU/mL.

### 2.2. Long-Term Exposure to TiO_2_ Affects the Cell Cycle

**Figure 2:**
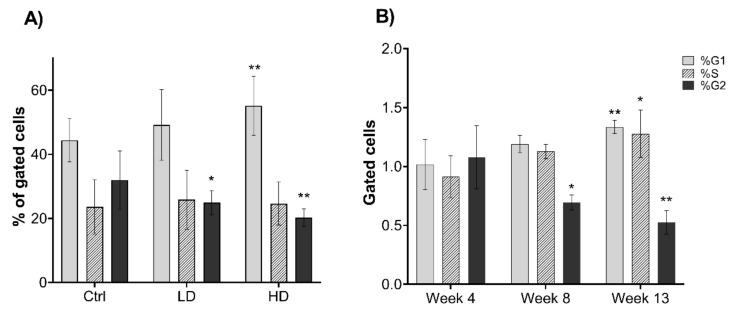
Effects of TiO_2_ exposure on cell cycle distribution. Cell cycle was analyzed by flow cytometry at 4, 8 and 13 weeks of continuous exposure. Single cells were gated. (**A**) Merged data of all weeks measured, presented as % of gated cells, (**B**) merged LD and HD data are shown as number of gated cells normalized to control cells (Ctrl), compared to week 4. Low dose (LD): 0.96 µg/cm^2^ and high dose (HD): 1.92 µg/cm^2^. Data indicate mean ± SEM, (A: *n* = 6, B: *n* = 4). * *p* < 0.05, ** *p* < 0.01 obtained from linear mixed model analysis.

Cell cycle was analyzed at 4, 8 and 13 weeks of continuous exposure to TiO_2_. Titanium dioxide exposure mediated a dose-dependent increase in the fraction of cells in G1 compared to control (Ctrl), which was statistically significant for the high dose (HD) group (*p* = 0.007). A concomitant decrease in the G2 fraction was found following TiO_2_ exposure at both low dose (LD) and HD (*p* = 0.043 and *p* = 0.003, respectively; Figure 2A). Furthermore, TiO_2_ exposure led to a significant increase in fraction of the cells at G1 (*p* = 0.008) and S-phase (*p* = 0.01) at week 13, and a decrease in G2-phase at week 8 (*p* = 0.01) and week 13 (*p* = 0.001) compared with week 4 (Figure 2B). Under our experimental conditions, TiO_2_ was not found to interfere with the cell cycle analysis by flow cytometry, as shown in Supplementary File S1: Figure S1.

### 2.3. Long-Term TiO_2_ Exposure Induces Multiple Cell Death Pathways

**Figure 3:**
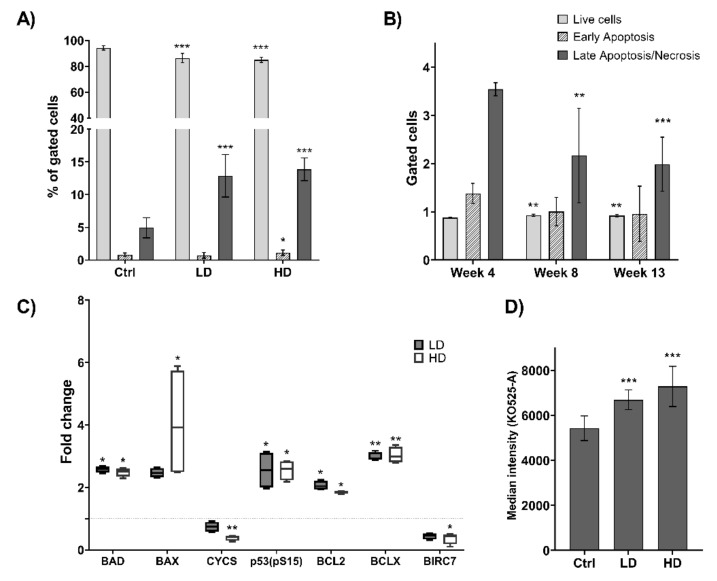
Effects of TiO_2_ exposure on cell death pathways. Effects on apoptosis and autophagy were analyzed after 4, 8 and 13 weeks of exposure to TiO_2_. Apoptosis was measured by flow cytometry using Annexin V and PI staining and illustrated as (**A**) percentage of gated cells of merged data from all weeks, compared to Ctrl, and (**B**) merged LD and HD data shown as the number of gated cells normalized to Ctrl, compared to week 4. (**C**) Data illustrate fold changes in the expression of apoptotic proteins measured after 13 weeks of exposure, compared to Ctrl. (**D**) Autophagy was measured by flow cytometry. Changes in autophagic vacuoles are illustrated as alterations in median fluorescent intensities. Data represent merged data of all weeks. Control (Ctrl), low dose (LD): 0.96 µg/cm^2^ and high dose (HD): 1.92 µg/cm^2^. Data indicate mean ± SEM, (*n* = 6 (**A**,**D**), *n* = 4 (**B**,**C**)). * *p* < 0.05, ** *p* < 0.01, *** *p* < 0.001 (Linear regression and linear mixed model).

In order to study effects of long-term TiO_2_ exposure on cell death pathways, levels of apoptosis and autophagy were investigated. Cumulative analysis of all time points after exposure demonstrated significant reduction of live cells at both LD and HD (*p* < 0.001) compared with Ctrl. This was concurrent with a significant increase in the number of late apoptotic/necrotic cells (*p* < 0.001). A small but significant increase (*p* = 0.037) in early apoptosis was also observed in cells exposed to HD of TiO_2_ (Figure 3A). Analysis at each exposure time revealed that late apoptosis/necrosis was more prominent at the earliest time point (4 weeks) compared with later time points (Figure 3B). After 13 weeks of exposure, expressions of 35 proteins involved in apoptosis were quantified (Supplementary File S1: Table S1). Seven of these were >1.5-fold up- or downregulated in TiO_2_ exposed cells compared with Ctrl (Figure 3C). A 2.6-fold (*p* = 0.030) increase in p53 phosphorylated at Ser15 was observed, which is known to induce proapoptotic proteins, including BAD and BAX. This was substantiated by an increase in BAD (LD: 2.5-fold and *p* = 0.021; HD: 2.4-fold and *p* = 0.024), BAX (LD: 2.5-fold and *p* = 0.080; HD: 4.1-fold and *p* = 0.030) as well as reduction in the antiapoptotic protein BIRC7 at HD (2.7-fold and *p* = 0.046; Figure 3C). However, a reduced level of CYCS, which is released to the cytosol during apoptosis was also observed, in addition to a significant increase in the antiapoptotic proteins BCL2 (LD: 2.1-fold and *p* = 0.025; HD: 1.9-fold and *p* = 0.037) and BCLX (BCL2L1) after treatment (LD: 3.0-fold and *p* = 0.003; HD: 3.0-fold and *p* = 0.003).

The levels of autophagy were also investigated at different times of TiO_2_ exposure. Although autophagy has generally been regarded a cell survival mechanism, it has also been shown to promote cell death. We observed an increase in autophagic vacuoles in LD and HD exposed cells (*p* < 0.001) compared with Ctrl (Figure 3D). This induction was seen at 4 weeks of exposure and remained throughout the exposure period (data not shown). Titanium dioxide did not interfere with the analysis of apoptosis and autophagy by flow cytometry at the doses used in this study, Supplementary File S1: Figures S2 and S3.

### 2.4. Effect of Long-Term TiO_2_ Exposure on Telomere Length

**Figure 4:**
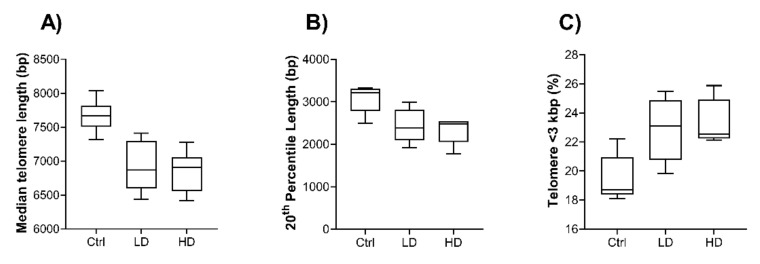
Effects of TiO_2_ exposure on telomere length. Telomere length was measured after 13 weeks of exposure by high-throughput Q-FISH. (**A**) Median telomere length measured in base pairs (bp). (**B**) Twentieth percentile telomere length measured in bp. (**C**) Percentage of telomeres with a length <3kbp. Control (Ctrl), low dose (LD): 0.96 µg/cm^2^ and high dose (HD): 1.92 µg/cm^2^. Box plots indicate median and 5–95 percentile (*n* = 10).

To determine if long-term exposure to TiO_2_ affects telomere length, a high-throughput Q-FISH technique was applied. Cells exposed to TiO_2_ for 13 weeks showed a trend of shorter telomere length at both doses compared with Ctrl (Figure 4). Median telomere length showed decreasing trend from 7682 bp in Ctrl to 6927 bp and 6847 bp in LD and HD, respectively (Figure 4A). Similarly, the 20th percentile length (bp) decreased from 3079 bp in Ctrl to 2448 bp and 2301 bp in LD and HD, respectively (Figure 4B). Exposed cells also showed higher percentage of telomere length < 3000 bp compared to Ctrl (Figure 4C). However, none of these changes in exposed and Ctrl cells were statistically significant.

### 2.5. Long-Term TiO_2_ Exposure Affects the Expression of Genome Maintenance Proteins

**Figure 5:**
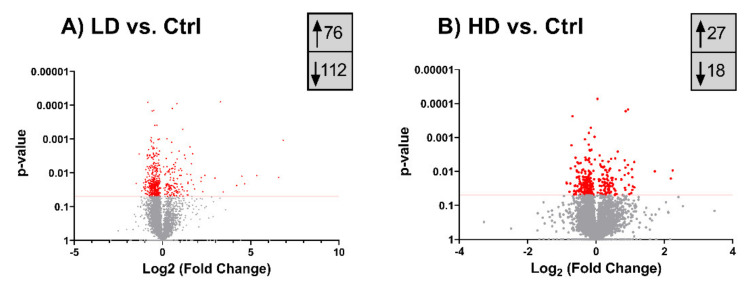
Differentially expressed proteins following 13 weeks TiO_2_ exposure. (**A**) Differentially expressed proteins of LD relative to Ctrl. (**B**) Differentially expressed proteins of HD relative to Ctrl. The horizontal red lines indicate a *p*-value cut-off of 0.05 (proteins with *p* ≤ 0.05 indicated as red dots). The actual numbers of significantly up- and downregulated proteins are boxed in grey. Control (Ctrl), low dose (LD): 0.96 µg/cm^2^ and high dose (HD): 1.92 µg/cm^2^.

Changes in protein expression after 13 weeks exposure to TiO_2_ are illustrated as volcano plots in Figure 5. Surprisingly, the number of differentially expressed proteins was higher in cells exposed at LD (188) than at HD (45). At LD, 76 proteins were significantly upregulated whereas 112 were downregulated (Figure 5A) while at HD 27 proteins were significantly upregulated and 18 downregulated (Figure 5B). Of these, 17 proteins were significantly (*p* < 0.050), > 1.5 fold regulated in both LD and HD (proteins with exact fold and *p*-values are shown in Supplementary File S1: Table S2). Interestingly, six out of seven upregulated proteins in this subset have been associated with cellular stress response mechanisms, including oxidative, DNA damage and replication stress responses. The most highly upregulated among these was GADL1 with fold changes of 22.5 (*p* = 0.013) and 4.7 (*p* = 0.009), for LD and HD respectively. NABP2 (LD: 7.9-fold, *p* = 0.014; HD: 4.6-fold, *p* = 0.016), MFAP1 (LD: 3.0-fold, *p* = 0.002; HD: 3.3-fold, *p* = 0.010), NUCKS1 (LD: 2.6-fold, *p* = 0.010; HD: 2.0-fold, *p* = 0.009), COPS9 (LD: 2.3-fold, *p* = 0.030; HD: 2-fold, *p* = 0.050), and CDK2NAIP (LD: 1.5-fold, *p* = 0.020; HD: 1.8-fold, *p* = 0.030) were also found upregulated in both doses. 

### 2.6. Analysis of Lipid Profiles after 13 Weeks of Exposure to TiO_2_

**Figure 6:**
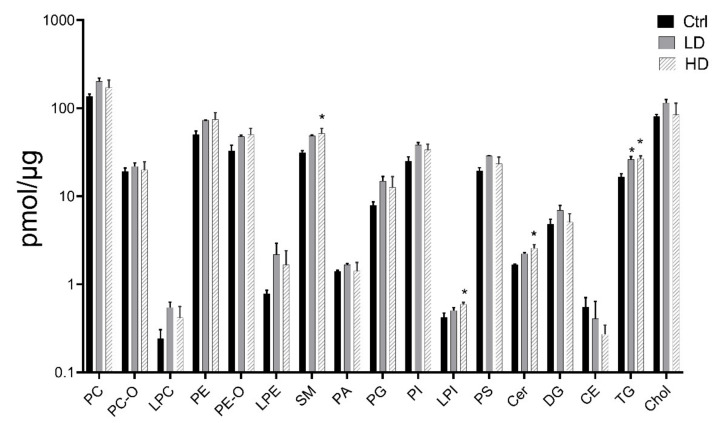
Effects of TiO_2_ exposure on lipid levels. Lipids were extracted and measured by shotgun lipidomics. Control (Ctrl), low dose (LD): 0.96 µg/cm^2^ and high dose (HD): 1.92 µg/cm^2^. Data indicate mean ± SEM, (*n* = 4). * *p* < 0.05, ** *p* < 0.01 (Linear mixed model).

The lipid composition of TiO_2_ exposed cells was measured by shotgun lipidomics after 13 weeks of exposure (Figure 6). The total lipid content in exposed cells increased compared to the Ctrl, from 430 pmol/µg protein to 630 pmol/µg protein in LD exposed cells, and 560 pmol/µg protein in HD exposed cells. Triacylglycerol (TG) was increased in both LD and HD exposed cells (*p* = 0.020 and *p* = 0.020, respectively). Moreover, sphingomyelin (SM, *p* = 0.040), lysophosphatidylinositol (LPI, *p* = 0.050), and ceramide (Cer, *p* = 0.020) levels were increased in cells exposed to the HD. A complete list over the analyzed lipid classes is shown in Supplementary File S1: Table S3.

## 3. Discussion

Most studies indicate that TiO_2_ exposure induces acute pulmonary inflammation [11,13], but our understanding of mechanisms driving long-term effects is limited. We have previously reported that long-term exposure of human pulmonary epithelial cells to TiO_2_ leads to colony formation in vitro [28], supporting that TiO_2_ exposure is associated with increased carcinogenic potential. Here, we investigated some key cellular pathways involved in lung fibrosis and carcinogenesis in pulmonary epithelial cells following long-term exposure to TiO_2_. Interestingly, we observed a significant shift in cell-cycle distribution of pulmonary cells after 13 weeks of exposure, with an enhanced number of G1 and S-phase cells and a concomitantly reduced number of cells in G2. This indicates that long-term exposure to TiO_2_ negatively affects entry into S-phase (G1/S checkpoint activation) as well as S-phase progression, both of which are indicative of genotoxic and replicative stress. However, the fraction of cells in G2 phase was reduced in long-term exposure to TiO_2_, which indicates nonadverse effect at the G2/M checkpoint. A potential explanation for this could be that DNA repair and replication fork protecting factors are mobilized, which reduces the number of DNA lesions passed on to G2/M but comes at the cost of delayed S-phase progression. In addition, specific proteins might contribute to attenuate G2/M checkpoint signaling. One such candidate would be HMGA2, which was fivefold upregulated in the cells treated at HD TiO_2_. Silencing of HMGA2 has been shown to mediate cell-cycle arrest in G2/M [32]. Upregulation of HMGA2 protein may also affect apoptosis through CASP3/9 and BCL2 [33]. Notably, CDK4, which primarily controls the G1/S checkpoint, is overexpressed in HBEC-3KT, was recently found to reciprocally activate p53 [34], offering a potential additional explanation to the differential enrichment in G1/S versus G2 by long-term TiO_2_-treatment.

The increase in apoptosis in TiO_2_ exposed cells, which was most prominent at the earliest time point (4 weeks), was concurrent with alterations in the expression of several pro- and antiapoptotic proteins. It has been previously shown that TiO_2_ exposure can induce apoptosis in different types of cells [21,35], however, the involved pathway(s) varies between different cell lines. In normal human cells, TiO_2_ exposure triggers apoptotic cell death through a ROS-dependent mechanism via BAX activation and upregulation of FAS [36]. Titanium dioxide exposure may also trigger a mitochondrial apoptotic pathway via CASP8/tBID [22]. Proapoptotic BAX and BAD are located in the cytosol and translocate to the mitochondria, and upregulation of these proteins results in CYCS release, which induces apoptotic stimuli. In agreement, we observed increased levels of BAX and BAD after TiO_2_ exposure. However, we found a concomitant downregulation of CYCS. It is generally accepted that release of CYCS from mitochondria is the point of commitment to apoptosis. Nevertheless, in some cells this is counteracted by proteasomal degradation of CYCS in the cytosol [37]. An upregulation of BCLX and BCL2 was also observed. These are traditionally regarded as antiapoptotic signals. It should be noted, that BCLX exists in several isoforms, of which BCLX(S) is proapoptotic, while BCLX(L) is antiapoptotic. As the analysis does not distinguish between the isoforms we cannot determine their individual contribution to the measured signal. The upregulation of BCL2 could indicate a possible induction of apoptosis via an alternative pathway such as TP53. In fact, our results showed that phospho-p53 (S15) was upregulated in both LD and HD exposed cells, potentially inducing apoptosis by multiple pathways.

Similar to apoptosis, autophagy is an important mechanism in regulation of cell death and upholding cellular homeostasis. It is known that autophagy can block apoptotic events. However, an induction in both processes was observed following TiO_2_. Interestingly, the signaling pathways regulating these processes are interlinked [38]. TP53, which is a potent inducer of apoptosis, can also increase the expression of DRAM1 and in turn induce autophagy [39]. Furthermore, c-Jun N-terminal kinase (JNK)-mediated phosphorylation of BCL2 may be a common pathway for regulation of autophagy and apoptosis [40]. It has previously been reported a link between nanoparticle exposure and autophagy, supporting our findings that TiO_2_ exposure may induce autophagy [41,42]. It has also been suggested that nanoparticles may induce autophagy through an oxidative stress mechanism [43]. In this study, TMBIM6 was found to be downregulated at HD. TMBIM6 is a BAX inhibitor 1 protein, a suppressor of apoptosis, and has a negative role in regulation of autophagy and autophagosome formation [44]. VAMP8 which is a SNAP Receptor protein involved in autophagy through direct control of autophagosome membrane fusion [45], was upregulated at LD. Among the upregulated proteins after long-term TiO_2_ exposure was FAM83A, which recently was shown to be a positive regulator of authophagy via TSPAN1 [46]. In support of our data, NOTCH1, which was downregulated following TiO_2_ exposure, was recently shown to be inversely correlated with autophagic flux [47]. In summary, these results demonstrate that long-term exposure to nanosized TiO_2_ may trigger alternative cell death pathways such as autophagy.

It is important to note that unique physicochemical properties and increased reactivity of nanoparticles increase the likelihood for their interference with photometric and fluorometric assays [48,49]. In the case of flow cytometric assays, nanoparticles may enhance or quench the fluorescence signals of the dyes or affect the measurements due to intrinsic fluorescence [50,51,52,53,54]. We therefore included controls to eliminate potential contribution of nanoparticle interference in the fluorometric assays. Using a spiked-in control to mimic a worst-case scenario, we did not detect interference of TiO_2_ with the utilized assays (Supplementary File S1: Figures S1–S3). Kroll et al., 2012 observed interference of TiO_2_ with in vitro toxicity assays at 50 µg/cm^2^ concentration but not below 10 µg/cm^2^ [49]. Li et al., showed that TiO_2_ particles emit fluorescence, but this was also dose-dependently decreasing with decreasing doses being highest at 400 µg/mL and lowest at 25 µg/mL [53]. Similarly, two other studies have shown that the interference of nanomaterials with flow cytometry assays is dose-dependent and is more likely to be significant at higher concentrations [50,51]. Thus, data from previous studies suggest that nanoparticle interference with different fluorometric assays likely occurs at particle concentrations significantly higher than those used in the current study, which is in agreement with our data showing no detectable interference with the fluorometric assays used here.

Changes in telomeres including shortening of telomeres are mainly associated with cellular senescence, and chronic disorders such as cancer and cardiovascular disease [55,56]. The mechanisms underlying this are poorly understood, but telomere shortening via oxidation of guanines in the telomeric regions has been associated with oxidative stress [57]. However, we observed a trend toward telomere length shortening after 13 weeks of exposure to TiO_2_. Titanium dioxide exposure has previously been shown to reduce telomere length [58]. Proteomic data revealed that LMNA protein, which is involved in telomere dynamics [59], was downregulated at LD. Moreover, single-strand binding protein 1, NABP2, which is associated with telomeres by forming a complex with TERT [60], was upregulated at both doses. Telomere dysfunction is associated with DNA damage checkpoint responses and cell cycle arrest [61,62] and activities TP53 and P16, which may induce cell senescence or apoptosis [62]. Similarly, we observed an increase in early and late apoptotic/necrotic cells in TiO_2_ exposed cells. This suggests that TiO_2_ exposure may affect telomere length, which could contribute to an activation of autophagic and apoptotic cell death mechanisms.

Proteomic data revealed an upregulation of GADL1, a multifunctional decarboxylase that is involved in protection against oxidative stress via production of carnosine peptides [63]. NABP2 relocates to replication forks during replication stress and is required for ATR- and CHEK1-mediated homologous recombination and restart of stalled replication forks [64]. Increased genome stress is further substantiated by upregulation of the spliceosome component MFAP1. A recent study demonstrated that MFAP1 along with its yeast homolog SPP381 are involved in regulation of a large number of genes involved in cell cycle regulation and the DNA damage response [65]. NUCKS1 is a chromatin associated RAD51AP paralog important for homologous recombination repair and genome stability [66]. A recent study demonstrated that NUCKS1 stimulates the ATPase activity of RAD54 and RAD51-RAD54-mediated strand invasion during homologous recombination repair [67]. COPS9 is part of the COP9 signalosome that removes the ubiquitin-like protein NEDD8 from cullins and thereby controls ubiquitinylation of proteins involved in the DNA damage response, reviewed in Hannss and Dubiel [68]. Finally, CDK2NAIP is part of the DNA damage response associated with replication stress and telomere shortening, and is upregulated in cells undergoing replicative or stress-induced senescence [36]. In addition to these, the DNA glycosylase MPG was significantly upregulated at both LD and HD. In DNA base-excision repair, MPG excises certain methylated bases such as 3-methyladenine and 7-methylguanine and was recently shown to coordinate DNA repair with gene expression [69]. We also found a subset of the downregulated proteins, which are factors associated with response to genotoxic stress, including apoptosis. The poly(ADP-ribose) glycohydrolase (PARG) hydrolyzes the ribose–ribose bonds of poly(ADP-ribose). In DNA damage response, poly(ADP)-ribosylation contributes to tether DNA-repair factors to chromatin and thus to ensure a robust DNA-repair response [70]. Downregulation of PARG would contribute to sustain this response. In summary, this strongly suggests that long-time exposure to low dose TiO_2_ nanoparticles induces genomic stress and activates multiple pathways involved in genomic maintenance.

Lipid composition is critical in pulmonary inflammatory diseases, as changes in lipid content play a role in development of COPD, cystic fibrosis, and asthma [71,72]. We and others have shown that nanoparticle exposure may also alter lipid composition [73,74]. TiO_2_ exposure has been linked to induced lipid peroxidation and cell membrane damage [75]. Our lipidomic analysis revealed an accumulation of total lipids in TiO_2_ exposed cells. The long-chain sphingolipids ceramide and sphingomyelin were dose-dependently increased following TiO_2_ exposure and can be associated with increased apoptosis and autophagy observed following long-term TiO_2_ exposure. These lipid classes are implicated in the regulation of cell growth, proliferation, differentiation, apoptosis and inflammatory responses, as increased levels of ceramide and sphingomyelin inhibit cell growth and induce apoptosis [76,77,78]. In addition to ceramide and sphingomyelin, several other lipid classes were accumulated following TiO_2_ exposure. Triacylglycerol was significantly increased at both LD and HD. Proteomic analysis revealed an upregulation of PON2 protein following TiO_2_ exposure. PON2 has been previously shown to be induced by increased triacylglycerol levels, which results in ROS induction in macrophages [79]. Furthermore, lysophosphatidylinositol, which was induced at HD, is important for several metabolic functions and may play a role in inflammation and cancer [80].

Congruently, our findings show that long-term TiO_2_ exposure results in enhanced G1 arrest and delayed S-phase progression accompanied by an induction in apoptosis and autophagy in bronchial epithelial cells. These changes correspond with alterations in pro- and antiapoptotic protein levels and accumulation of several lipid classes involved in cell stress and apoptosis. These findings are further supported by proteomic data showing an increase in proteins involved in genotoxic stress and genomic maintenance pathways following long-term TiO_2_ exposure. The study is limited in the number of biological replicates included, but altogether suggests a role of genotoxic stress response and induction of cell death pathways in long-term effects of TiO_2_. These data provide new mechanistic insight into TiO_2_-induced pulmonary effects and further investigation is warranted in order to confirm the importance of these signaling events in TiO_2_-induced chronic respiratory diseases such as fibrosis and carcinogenesis.

## 4. Materials and Methods

### 4.1. Particle Dispersion and Characterization

Titanium dioxide (NM-62002a) obtained from the EU Joint Research Centre (Ispra, Italy) is one test material selected by the OECD working party for manufactured nanomaterials [81]. The NANOGENOTOX dispersion protocol was applied for particle dispersion and cell exposure, as previously described [82]. Briefly, 2.56 mg/mL stock solution was prepared by a mixture of 15.36 mg dry TiO_2_ powder (prewetted in 0.5% (*v/v*) EtOH in scintillation vials and a dispersion medium of 0.05% (*v/v*) BSA (Sigma-Aldrich; St. Louis, MO, USA) and MilliQ-H_2_O. The mixture was sonicated (Branson Sonifier S-450D) for 16 min at 10% amplitude on ice. Titanium dioxide stock solutions were prepared immediately prior to exposure. The TiO_2_ nanomaterial used in this study has previously been well characterized and reported by Rasmussen, et al. [83], with a reported primary diameter of 26 ± 10 (nm ± SE) and a Benchmark Z-size of 234 ± 4 nm [84]. Further analysis of characterization was performed by our group with scanning electron microscopes (SEM), and the hydrodynamic diameter of particles in dispersion media and in exposure media were measured by dynamic light scattering (DLS) (Zetasizer Nano, Malvern Instruments, Ltd.; Worcestershire, UK) at the time of exposure (0 h) and after 72 h. The levels of endotoxin were assessed by kinetic chromogenic limulus amebocyte lysate (LAL) assay according to the manufacturer’s instructions (Lonza, Basel, Switzerland).

### 4.2. Long-Term Cell Exposure

Human bronchial epithelial cells 3KT (HBEC-3KT, ATCC^®^ CRL-4051™) were used in this study. HBEC-3KT cells are normal nontumorigenic cells immortalized with CDK4 and hTERT. The cells were maintained in a serum-free 1:1 mixture of LHC-9 (Gibco, Thermo Fisher Scientific; Waltham, MA, USA) and RPMI-1640 (Thermo Fisher Scientific) medium containing 100 units/mL penicillin and 100 µg/mL streptomycin, in a humidified 5% CO_2_ atmosphere at 37 °C. HBEC-3KT cells were seeded at 2.5 × 10^5^ cells/plate in 15 cm plate (Sarstedt; Nümbrecht, Germany; growth area: 152 cm^2^), and exposed twice per week to TiO_2_ at concentrations; 0.96 µg/cm^2^, designed as low-dose (LD) and 1.92 µg/cm^2^, designed as high-dose (HD). Control (Ctrl) cells were only exposed to the vehicle dispersion solution. The cells were exposed to TiO_2_ twice a week for 13 weeks continuously and subcultured once per week. Doses were selected based on the recommended exposure limit (REL) of 0.3 mg/m^3^ for ultrafine TiO_2_ proposed by NIOSH, Cincinnati, OH, USA [85]. The LD dose corresponded to a maximum lifetime dose accumulated by a worker 5 workdays/week, 48 weeks/year for 42 years. Assumption of an accumulation of breathing volume of 3 m^3^ in 8 h, a lung surface area of 140 m^2^ and around 50% alveolar deposition efficiency for 10–30 nm TiO_2_, was made for calculations. The selected HD is two times the LD.

### 4.3. Flow Cytometry

After 4, 8 and 13 weeks of exposure, we determined cell cycle, apoptosis and autophagy using flow cytometry. Cell cycle was determined by propidium iodide (PI) staining. In short, the cells were fixed with 70% ice-cold ethanol for 30 min. The fixed cells were preincubated with 50 µL of 100 µg/mL RNase, to degrade RNA, DNA was stained with 100 µL of 50 µg/mL PI (Sigma-Aldrich) in cell staining buffer (BioLegend; San Diego, CA, USA). Analyses were performed on a linear scale. Single cells were gated and included in the analysis.

Apoptotic cells were detected by Annexin V—APC conjugate and necrotic cells by PI staining (Invitrogen, Thermo Fisher Scientific). Annexin V is a protein with a strong affinity for phosphatidylserine, which is released to the cell surface after initiating apoptosis. Briefly, cell media was collected for floating apoptotic and necrotic cells, and adherent cells were collected by trypsinization. Cells were left at 37 °C for half an hour to recover, before staining with Annexin. For induction of apoptosis and necrosis, cells were heat-shocked by incubation for 5 min at 56 °C. Unstained sample was used as negative control. The positive and negative controls were used to set the gating strategy. Cells that appeared in the Annexin V/PI and PI positive were merged together and presented as one group.

Autophagy, i.e., fluorescent intensity of autophagic vacuoles, was analyzed by CYTO-ID^®^ Autophagy detection kit (Enzo Biochem, Farmingdale, NY, USA). Briefly, adherent HBEC-3KT cells were trypsinized, stained with CYTO-ID^®^ Green stain solution and buffer solution, and incubated at RT for 30 min in the dark. After treatment, cells were washed once with assay buffer and run with KO525 filter. For positive control, cells were added a combination of 500 mM Rapamycin and 10 µM Chloroquine (included by the supplier) and incubated for 16–18 h before staining.

For all experiments, 1 × 10^6^ cells/mL were used to assess the different end points using standard protocols provided by the manufacturers unless otherwise stated. All samples were filtered after trypsinization using 70 µm cell strainers (VWR, Radnor, PA, USA) to remove larger cell aggregates and particle aggregates/agglomerates. Samples were run using CytoFLEX Flow Cytometer (Beckman Coulter, Brea, CA, USA), with 10,000 events recorded for further analysis. Gating and analyses were performed using the FSC express 7 Flow Cytometry software (De Novo, Glendale, CA, USA). In the utilized gating strategy, small fragments and nanoparticles were gated out in the FSC/SSC scatter plot and excluded from further analysis.

Analysis of TiO_2_ interference with the flow cytometric assays was performed essentially as described in Bohmer et al., 2018 [50]. In short, untreated cells and the positive control cells were spiked-in with TiO_2_ directly in the staining solution. These samples were compared to unspiked controls. The spiked-in controls represent a worst-case scenario where 100% deposition and uptake of the highest applied nanomaterial dose is assumed (1.92 µg/cm^2^), Supplementary File S1. Unstained controls with and without spiked-in TiO_2_ were also performed (data not shown).

### 4.4. Apoptosis Array

Using Proteome Profiler™ Human Apoptosis Array Kit (R&D Systems, Minneapolis, MI, USA), 35 apoptosis-related proteins were analyzed after 13 weeks of exposure to TiO_2_. Cell lysis, protein isolation and detection were carried out following the manufacturer’s protocol. An amount of 300 µg protein was applied on protein array membranes and signals were detected on an AI600RGB imaging system (GE Healthcare, Chicago, IL, USA). Intensity signals were quantified with ImageQuant TL (GE Healthcare).

### 4.5. Telomere Length

Analysis of median telomere length following 13 weeks of TiO_2_ exposure was performed at Life Length Company (Madrid, Spain) using high-throughput Q-FISH technique. Briefly, 15,000 cells were seeded in 384-well plates with five replicates of each sample. Cells were fixed with MeOH/AcOH (3:1, *v/v*), and DNA stained with DAPI. Quantitative image acquisition and analysis were performed on a High Content Screening Opera System (Perkin Elmer, Waltham, MA, USA), using the Acapella software, Version 1.8 (Perkin Elmer). Telomere length distribution and median telomere length were calculated with Life Length’s proprietary algorithms [86].

### 4.6. Proteomics

Quantitative LC-MS/MS using EASY-nLC 1000 UHPC system was performed essentially as previously described [73]. Briefly, proteins were precipitated by MeOH/CHCl_3_. LC-MS/MS analysis was performed on an EASY-nLC 1000 UHPC system with C18 columns interfaced with an Orbitrap Elite mass spectrometer via a Nanospray Flex ion source using a data-dependent strategy. A total of 5483 protein groups were identified, of which 4819 were quantified across all samples. Only proteins with 100% identification (FDR < 0.01 using target-decoy protein) in at least one group were included in the analysis. Further, only proteins that were detected in all three samples (Ctrl, LD and HD), were considered for the comparative analysis. Statistical analysis was carried out using Student’s t-test, and only proteins with *p*-value < 0.05 and fold change > 1.5 were selected for further analysis. Given the small number of biological replicates, we refrained from inferring rigorous statistical analysis. The complete dataset including search results has been deposited to the ProteomeXchange Consortium via the PRIDE partner repository [87], with the project ID PXD025423. The lists of differentially expressed proteins in LD vs. Ctrl and HD vs. Ctrl were subjected to String analysis, and the values of specifically enriched KEGG pathways were mapped using Pathview via Bioconductor version 3.11 [88].

### 4.7. Lipidomics

Lipid quantification was performed by shotgun mass spectrometry as previously described [73]. Briefly, control cells and cells exposed for 13 weeks to TiO_2_ were homogenized in isopropanol and concentration of the proteins were measured by Pierce BCA Protein Assay Kit (Pierce, Thermo Fisher Scientific). An amount of 50 µg of total protein was extracted with MTBE/MeOH (10:3), the upper phase was collected and reconstituted in MeOH/CHCl_3_ (2:1). Analysis of mass spectrometry was performed using Q Exactive instrument (Thermo Fischer Scientifc). Lipids were identified by LipidXplorer software [89]. Supplementary File S2 includes LipidXplorer output file. Intact lipid masses with mass accuracy better than 5 ppm were identified, and lipids were quantified by comparing molecular ions isotopic corrected abundances with the abundances of internal standards of the same lipid class.

### 4.8. Statistical Anaysis

Due to practical limitations in performing the long-term exposures, experiments were only performed once but with two biological replicates in each exposure group. Flow cytometry data were analyzed using a linear regression statistical model. Data measured in week 4, 8 and 13 were merged to achieve better power for the statistical analysis. The effect of LD and HD with respect to Ctrl was analyzed first by including the exposure group as covariate and adjusting for exposure time. In comparison of weeks, the LD and HD measurements for each time (week) were normalized to the average values of the Ctrl’s. The effect of time exposure (week) was analyzed by assuming a common effect of LD and HD cells, and by including week as a covariate. Apoptosis, lipidomic and telomere length measurements were only conducted at the longest exposure time (week 13) and analyzed using a linear mixed model. Exposure group was included as a fixed effect, and experiment as a random intercept. For analysis of proteomic data, a Student’s t-test was performed. All analyses were done in R, version 3.6.3, package lme4 and lmerTest. Figures were made using GraphPad Prism 8 (GraphPad Software, San Diego, CA, USA). For all statistical analyses, a *p*-value ≤ 0.05 was considered statistically significant.

## Data Availability

Proteomic data is available at ProteomeXchange Consortium via the PRIDE partner repository, with the project ID PXD025423. Lipidomic data and other data are available upon request to corresponding author.

## Supplementary Materials

The following are available online at https://www.mdpi.com/article/10.3390/ijms22105349/s1, Supplementary File S1 includes: Table S1: Apoptosis-related proteins measured after 13 weeks of exposure to TiO_2_, Table S2: Altered proteins commonly regulated in LD/HD exposed cells vs. control, Table S3: Lipid composition measured after 13 weeks of exposure to TiO_2_, Figure S1: Assessment TiO_2_ nanoparticle interference with the cell cycle analysis using flow cytometry, Figure S2: Assessment TiO_2_ nanoparticle interference with the analysis of apoptosis by flow cytometry, and Figure S3: Assessment TiO_2_ nanoparticle interference with the analysis of autophagy by flow cytometry. Supplementary File S2 includes LipidXplorer output file (csv).
