# Supplementary material for: Long-Term Exposure to Nanosized TiO2 Triggers Stress Responses and Cell Death Pathways in Pulmonary Epithelial Cells
Source: Int J Mol Sci. 2021 May 19;22(10):5349. doi: 10.3390/ijms22105349 (PMC8161419; doi:10.3390/ijms22105349)
Supplement: Supplementary file 1 [file ijms-22-05349-s001.zip › Supplementary file 1.pdf]

Supplementary file 1: Tables

Table S1: Apoptosis-related proteins measured after 13 weeks of exposure to TiO_2. Significant proteins with p-values <0.05 are marked in *italics*. Arrows indicate up- and downregulation.

Gene symbol	Protein names	LD		HD	
		Fold change	p-value	Fold change	p-value
BAD	BCL2 Associated Agonist Of Cell Death	2.461↑	*0.021*	2.371↑	*0.024*
BAX	BCL2 Associated X, Apoptosis Regulator	2.469↑	0.080	4.053↑	*0.033*
BCL2	BCL2 Apoptosis Regulator	2.064↑	*0.025*	1.852↑	*0.037*
BCLX (BCL2L)	BCL2 Like 1	2.980↑	*0.003*	3.030↑	*0.003*
CASP3	Pro-Caspase-3	1.170	0.172	1.148	0.212
	Cleaved Caspase-3	1.488	0.050	1.320	0.200
CAT	Catalase	1.207	0.560	1.361	0.895
BIRC2	Baculoviral IAP Repeat Containing 2	1.073	0.691	1.139	0.695
BIRC3	Baculoviral IAP Repeat Containing 3	1.184	0.444	1.055	0.837
BIRC5	Baculoviral IAP Repeat Containing 5	1.028	0.798	1.120	0.933
BIRC7	Baculoviral IAP Repeat Containing 7	2.271↓	0.094	2.682↓	*0.046*
CLSPN	Claspin	1.205	0.500	1.003	0.920
CLU	Clusterin	1.161	0.333	1.339	0.102
CYCS	Cytochrome c	1.344↓	0.140	2.615↓	*0.008*
TNFRSF10A	Tumor necrosis factor receptor superfamily member 10A	2.107↑	0.156	2.049↑	0.165
TNFRSF10B	Tumor necrosis factor receptor superfamily member 10B	1.220	0.524	1.015	0.901
FADD	Fas Associated Via Death Domain	1.582↑	0.193	1.668↑	0.151
FAS	Fas Cell Surface Death Receptor	1.220	0.456	1.123	0.625
HIF1A	Hypoxia Inducible Factor 1 Subunit Alpha	1.272	0.384	1.457	0.197
HMOX1	Heme Oxygenase 1	1.352	0.140	1.139	0.442
HMOX2	Heme Oxygenase 2	1.075	0.664	1.014	0.842
HSPB1	Heat Shock Protein Family B (Small) Member 1	1.145	0.494	1.038	0.777
HSPD1	Heat Shock Protein Family D (Hsp60) Member 1	1.377	0.186	1.229	0.348
HSPA4	Heat Shock Protein Family A (Hsp70) Member 4	1.118	0.549	1.033	0.857
HTRA2	HtrA Serine Peptidase 2	1.161	0.618	1.611	0.133
PON2	Paraoxonase 2	1.595	0.088	1.672	0.073
CDKN1A	Cyclin Dependent Kinase Inhibitor 1A	1.465	0.224	1.745	0.119
CDKN1B	Cyclin Dependent kinase Inhibitor 1B	1.181	0.536	1.328	0.368
TP53	Phosphoprotein p53 (Ser15)	2.556↑	*0.031*	2.556↑	*0.030*
	Phosphoprotein p53 (Ser46)	1.280	0.266	1.133	0.519
	Phosphoprotein p53 (Ser392)	1.994	0.134	1.614	0.237
	Phosphoprotein Rad17 (Ser635)	1.042	0.780	1.136	0.676
DIABLO	Diablo IAP-Binding Mitochondrial Protein	1.490	0.144	1.439	0.163
TNFRSF1A	TNF Receptor Superfamily Member 1A	1.247	0.291	1.070	0.668
XIAP	X-Linked Inhibitor Of Apoptosis	1.064	0.765	1.475	0.088

Table S2: Altered proteins commonly regulated in LD/HD exposed cells vs. control. Quantitative proteomic analyses were conducted after 13 weeks of exposure to TiO_2. Arrows indicate up- and downregulation.

Gene symbol	Protein names	LD		HD	
		Fold change	p-value	Fold change	p-value
AKAP10	A-Kinase Anchoring Protein 10	2.500↓	0.003	1.504↓	0.047
B4GALT5	Beta-1,4-Galactosyltransferase 5	1.772↓	0.017	1.538↓	0.027
CDKN2AIP	CDKN2A Interacting Protein	1.518↑	0.024	1.813↑	0.034
COPS9	COP9 Signalosome Subunit 9	2.345↑	0.034	1.958↑	0.046
COX5B	Cytochrome C Oxidase Subunit 5B	1.606↓	0.007	1.684↓	0.023
GADL1	Glutamate Decarboxylase Like 1	22.517↑	0.013	4.720↑	0.009
HSD17B8	Hydroxysteroid 17-Beta Dehydrogenase 8	1.648↓	0.038	1.525↓	0.045
MARK3	Microtubule Affinity Regulating Kinase 3	2.223↑	0.001	1.616↓	0.000
MFAP1	Microfibril Associated Protein 1	2.950↑	0.002	3.293↑	0.010
NABP2	Nucleic Acid Binding Protein 2	7.898↑	0.014	4.556↑	0.016
NOTCH1	Notch Receptor 1	1.638↓	0.021	1.822↓	0.021
NUCKS1	Nuclear Casein Kinase And Cyclin Dependent Kinase Substrate 1	2.607↑	0.011	2.069↑	0.009
PARG	Poly(ADP-Ribose) Glycohydrolase	2.101↓	0.020	1.813↓	0.022
POLR3C	RNA Polymerase III Subunit C	1.560↓	0.003	1.521↓	0.026
RPS28	Ribosomal Protein S28	2.056↑	0.021	1.782↑	0.005
TPM1	Tropomyosin 1	2.231↑	0.048	1.528↓	0.007
TSR3	TSR3 Ribosome Maturation Factor	1.633↓	0.029	1.561↓	0.038

Table S3: Lipid composition measured after 13 weeks of exposure to TiO_2. Significantly regulated lipid classes with p-values <0.05 are marked in *italics*.

Lipid		Ctrl	LD		HD	
Symbol	Name	pmol/µg	pmol/µg	p-value	pmol/µg	p-value
PC	Phosphatidylcholine	136.400	202.060	0.128	171.714	0.357
PC-O	Ether-linked phosphatidylcholine	19.100	21.916	0.596	19.945	0.941
LPC	Lysophosphatidylcholine	0.200	0.546	0.105	0.419	0.292
PE	Phosphatidylethanolamine	50.500	73.082	0.141	74.858	0.120
PE-O	Ether-linked phosphatidylethanolamine	33.000	47.960	0.157	50.416	0.113
LPE	Lyso-phosphatidylethanolamine	0.801	2.182	0.161	1.671	0.327
SM	Sphingomyelin	31.600	48.750	0.053	51.775	*0.035*
PA	Phosphatidic acid	1.400	1.676	0.386	1.419	0.990
PG	Phosphatidylglycerol	7.900	14.894	0.127	12.653	0.254
PI	Phosphatidylinositol	25.300	38.416	0.070	33.921	0.178
LPI	Lysophosphatidylinositol	0.400	0.505	0.257	0.592	*0.050*
PS	Phosphatidylserine	19.600	28.778	0.073	23.580	0.351
Cer	Ceramide	1.700	2.241	0.050	2.577	*0.015*
DG	Diacylglycerol	4.900	6.967	0.201	5.120	0.955

CE	Cholesteryl esters	0.500	0.407	0.404	0.272	0.185
TG	Triacylglycerol	16.600	26.281	*0.020*	26.796	*0.017*
Chol	Cholesterol	80.500	115.067	0.231	84.826	0.900

Supplementary file 1: Figures

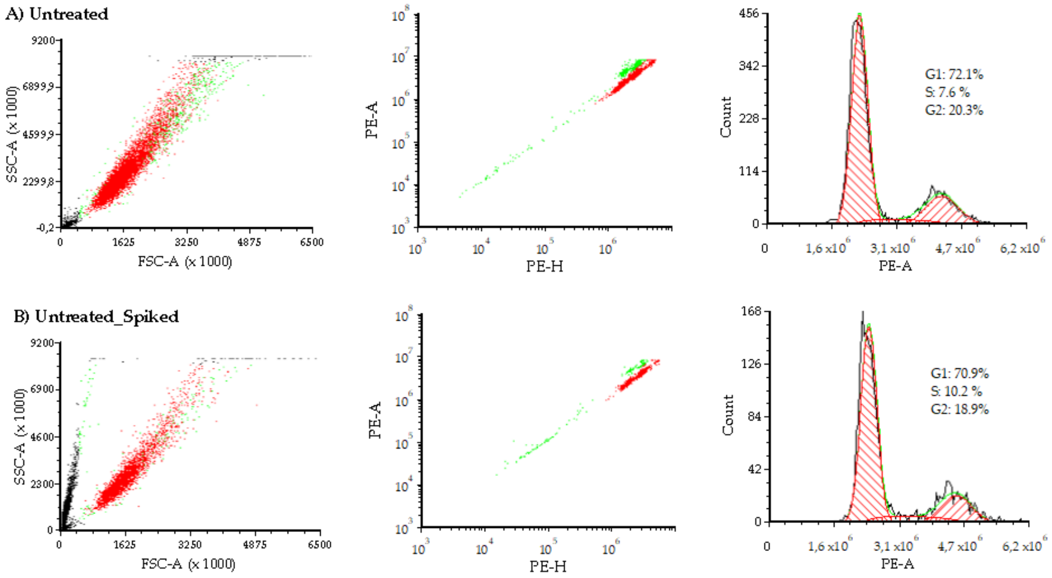

Figure S1. Assessment TiO_2 nanoparticle interference with the cell cycle analysis using flow cytometry. Representative scatter plots and histogram of untreated cells (A) and untreated cells spiked with TiO_2 (B).

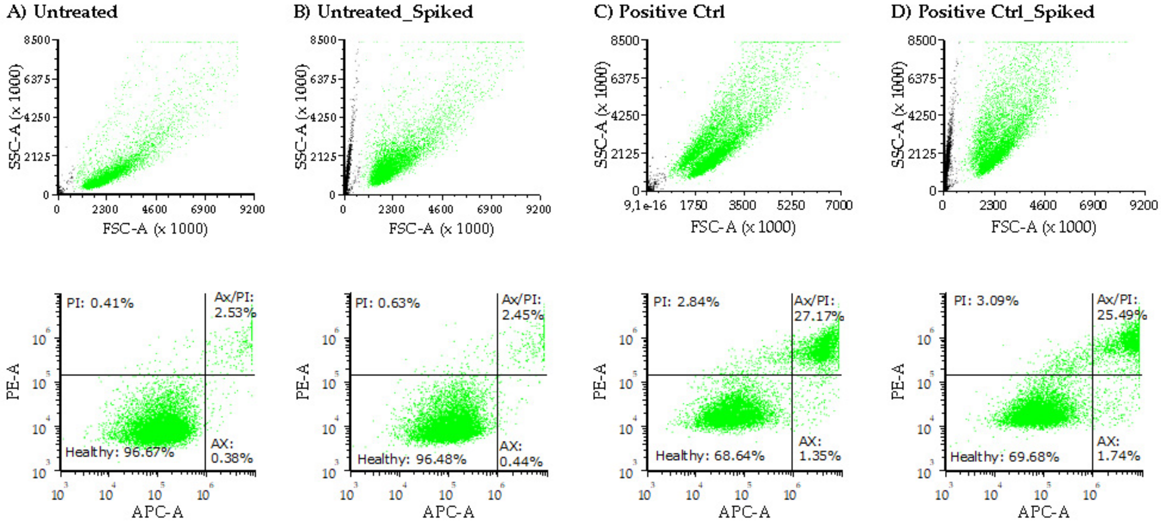

Figure S2. Assessment TiO_2 nanoparticle interference with the analysis of apoptosis by flow cytometry. Representative scatter plots of untreated cells (A), untreated cells spiked with TiO_2 (B), positive control (Ctrl) i.e. cells heat shocked at 56°C for 5 min (C) and positive control spiked with TiO_2 (D). PI: propidium iodide and AX: Annexin V.

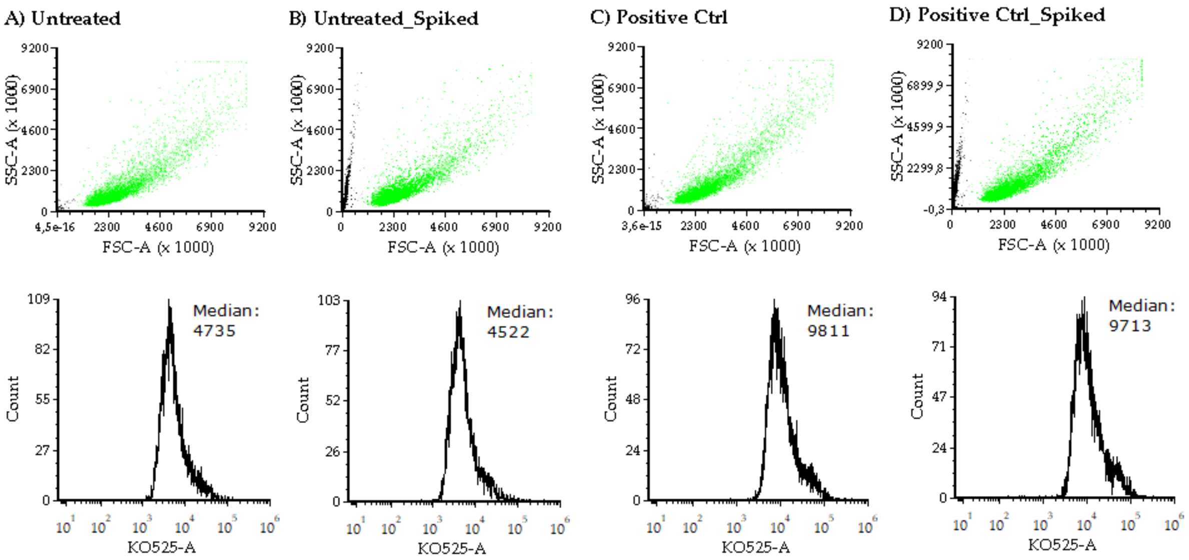

Figure S3. Assessment TiO_2 nanoparticle interference with the analysis of autophagy by flow cytometry. Representative scatter plot and histogram of untreated cells (A), untreated cells spiked with TiO_2 (B), positive control i.e. cells treated with 500 mM Rapamycin and 10 μM Chloroquine (C) and positive control spiked with TiO_2 (D).
