# Supplementary figures and images for: Long-Term Exposure to Nanosized TiO2 Triggers Stress Responses and Cell Death Pathways in Pulmonary Epithelial Cells
Source: Int J Mol Sci. 2021 May 19;22(10):5349. doi: 10.3390/ijms22105349 (PMC8161419; doi:10.3390/ijms22105349)

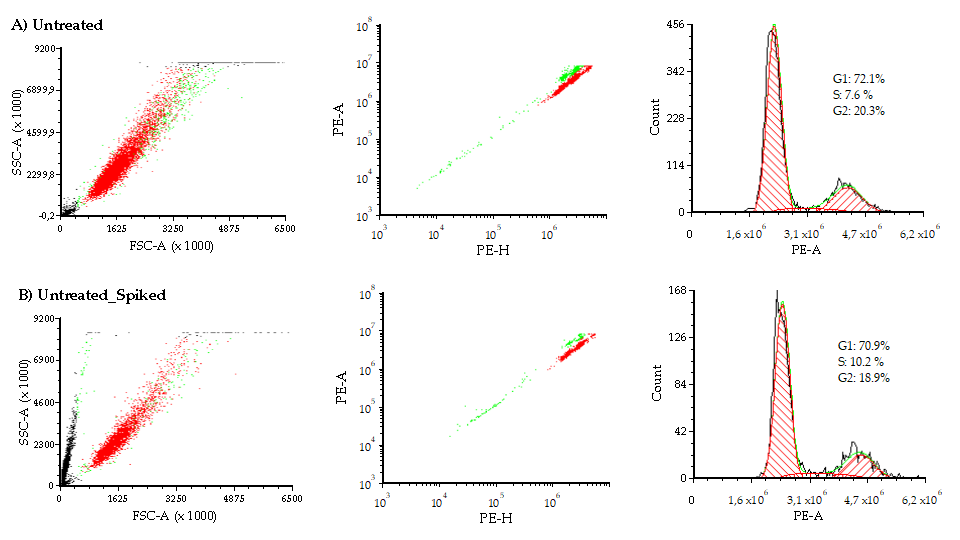

Supplement: Supplementary file 1 [file ijms-22-05349-s001.zip › Supplementary Figures/Figure S1. Assessment TiO2 nanoparticle interference with the cell cycle analysis using flow cytometry. .tif]

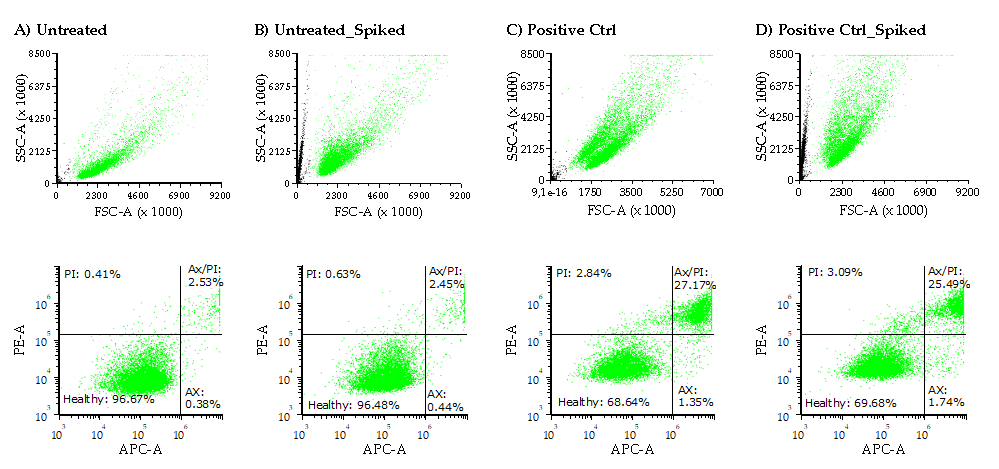

Supplement: Supplementary file 1 [file ijms-22-05349-s001.zip › Supplementary Figures/Figure S2. Assessment TiO2 nanoparticle interference with the analysis of apoptosis by flow cytometry.tif]

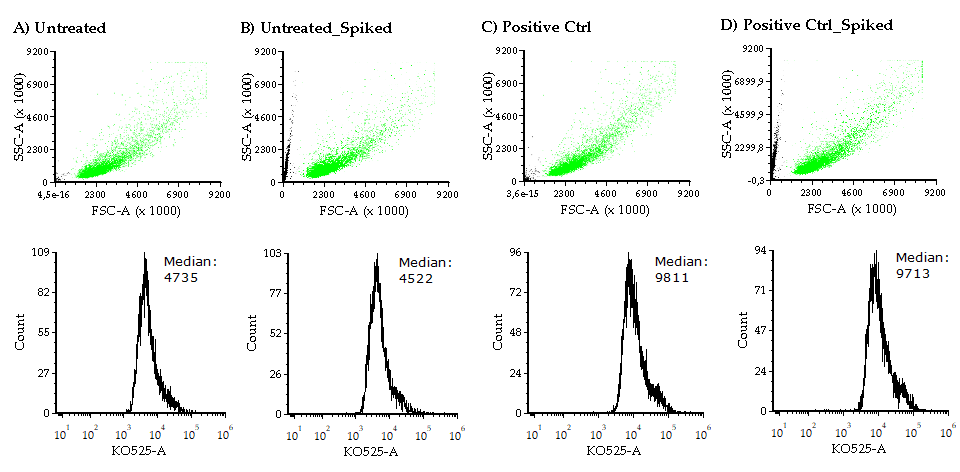

Supplement: Supplementary file 1 [file ijms-22-05349-s001.zip › Supplementary Figures/Figure S3. Assessment TiO2 nanoparticle interference with the analysis of autophagy by flow cytometry.tif]
